# Systematic evaluation of spatial transcriptomic annotation methods reveals conserved tumor microenvironment programs in NSCLC

**DOI:** 10.3389/fbinf.2026.1880333

**Published:** 2026-08-20

**Authors:** Mingke Wu, Yihan Zhou, Dingjie Xu

**Affiliations:** 1 Department of Immunology, School of Basic Medical Sciences, Peking University Beijing, Beijing, China; 2 Department of Pharmacology, School of Medicine, Yale University, New Haven, CT, United States

**Keywords:** cell-type annotation, non-small cell lung cancer (NSCLC), spatial transcriptomics, transcriptome pattern, tumor microenvironment

## Abstract

**Introduction:**

Spatial transcriptomics (ST) enables high-resolution mapping of cellular heterogeneity in tumor microenvironments, but downstream cell-type annotation remains highly method-dependent.

**Methods:**

Here, we systematically evaluated four representative annotation frameworks—Seurat, CARD, RCTD, and SPOTlight—across 45 non-small cell lung cancer (NSCLC) ST samples from 10×Visium comprising 328,561 spots. We performed a comprehensive cross-method assessment across multiple analytical dimensions, including cell-type composition, inter-method agreement, spatial architecture, cell-cell interaction patterns, and functional pathway activity.

**Results:**

While all methods captured broad tissue organization, substantial variability emerged in inferred cellular composition, spatial clustering, and functional states. CARD and RCTD demonstrated the highest overall concordance across abundance estimation and spatial structure, whereas Seurat and SPOTlight displayed greater method-specific divergence. Functional analyses further revealed method-specific biological programs, with CARD and RCTD better capturing stromal and myeloid identity. Importantly, beyond these differences, cross-method consensus analysis identified robust spatially conserved biological programs across multiple layers, including gene-level signatures, transcription factor activity, and pathway-level reprogramming characterized by enhanced metabolic and translational activity in tumor-proximal regions. These transcriptional signatures were further supported by independent single-cell RNA sequencing datasets, suggesting their biological relevance.

**Discussion:**

Overall, our study demonstrates that while no single annotation method is universally optimal, multi-method integration enables the identification of robust multi-layer spatial regulatory programs and provides a more reliable framework for interpreting ST data in complex tumor ecosystems.

## Introduction

Spatial transcriptomics (ST) technologies have enabled the simultaneous measurement of gene expression and spatial localization within intact tissues, providing unprecedented insights into tumor microenvironment (TME) organization (Janesick et al., 2023). In non-small cell lung cancer (NSCLC), ST has been widely used to investigate cellular heterogeneity, tumor-immune interactions, and spatially organized functional states that cannot be resolved by single-cell RNA sequencing alone (De Zuani et al., 2024). However, accurate interpretation of ST data critically depends on reliable cell-type annotation, which remains a major computational challenge (Fang et al., 2023).

To address this issue, a variety of computational methods have been developed for ST cell-type deconvolution and annotation. These methods can be broadly categorized into integration-based approaches (e.g., Seurat label transfer) (Hao et al., 2024), statistical mixture models (e.g., RCTD) (Cable et al., 2022), spatially aware frameworks (e.g., CARD) (Ma and Zhou, 2022), and marker-driven matrix factorization methods (e.g., SPOTlight) (Elosua-Bayes et al., 2021). Although each method has been independently validated, their underlying assumptions differ substantially, including how they model spatial dependence, transcriptional similarity, and cellular heterogeneity (Huang et al., 2024). As a result, the consistency and biological reliability of these methods remain unclear, particularly in complex tumor tissues such as NSCLC (Fu et al., 2025; Wang et al., 2023; Luo et al., 2024).

Previous studies have primarily focused on benchmarking individual methods or developing new algorithms, but systematic comparisons across multiple annotation frameworks NSCLC spatial transcriptomics remain limited (You et al., 2024; Long et al., 2023; Du et al., 2025). In particular, it is still unclear how method-dependent differences may influence biological interpretations of the NSCLC tumor microenvironment, including spatial organization of tumor and immune cells, intercellular interactions, and functional pathway activity, all of which are critical for understanding tumor progression and therapeutic response in lung cancer (Wu et al., 2025; An et al., 2025; Atta and Fan, 2021).

Here, we systematically compare four representative annotation methods: Seurat, CARD, RCTD, and SPOTlight across 45 NSCLC spatial transcriptomics samples. We evaluate how different computational frameworks influence downstream biological interpretation at multiple levels, including cell-type composition, spatial tissue architecture, tumor-immune organization, and functional pathway activity.

Importantly, beyond method benchmarking, we further integrate results across approaches to identify cross-method consensus signals, enabling the extraction of robust spatial gene expression programs that are less sensitive to algorithm-specific biases and more likely to reflect conserved biological structure. This integrative strategy highlights how computational variability can be leveraged rather than ignored and establishes a more reliable framework for interpreting tumor microenvironment architecture and identifying reproducible spatial programs in lung cancer.

## Results

### Global comparison reveals method-dependent shifts in cell-type composition

To systematically compare the performance of four cell-type annotation methods on NSCLC spatial transcriptomics (ST) data, we collected a total of 45 NSCLC Visium samples, including 20 samples from human non-small-cell lung cancer data (De Zuani et al., 2024), and 25 samples from GSE307534 (selected tumor site slides). As a single-cell RNA-sequencing (scRNA-seq) reference, we integrated lung cancer-related data from the integrated Human Lung Cell Atlas (https://data.humancellatlas.org/hca-bio-networks/lung/atlases/lung-v1-0) (Sikkema et al., 2023), totaling 114,549 cells classified into 10 major cell types.

**FIGURE 1:**
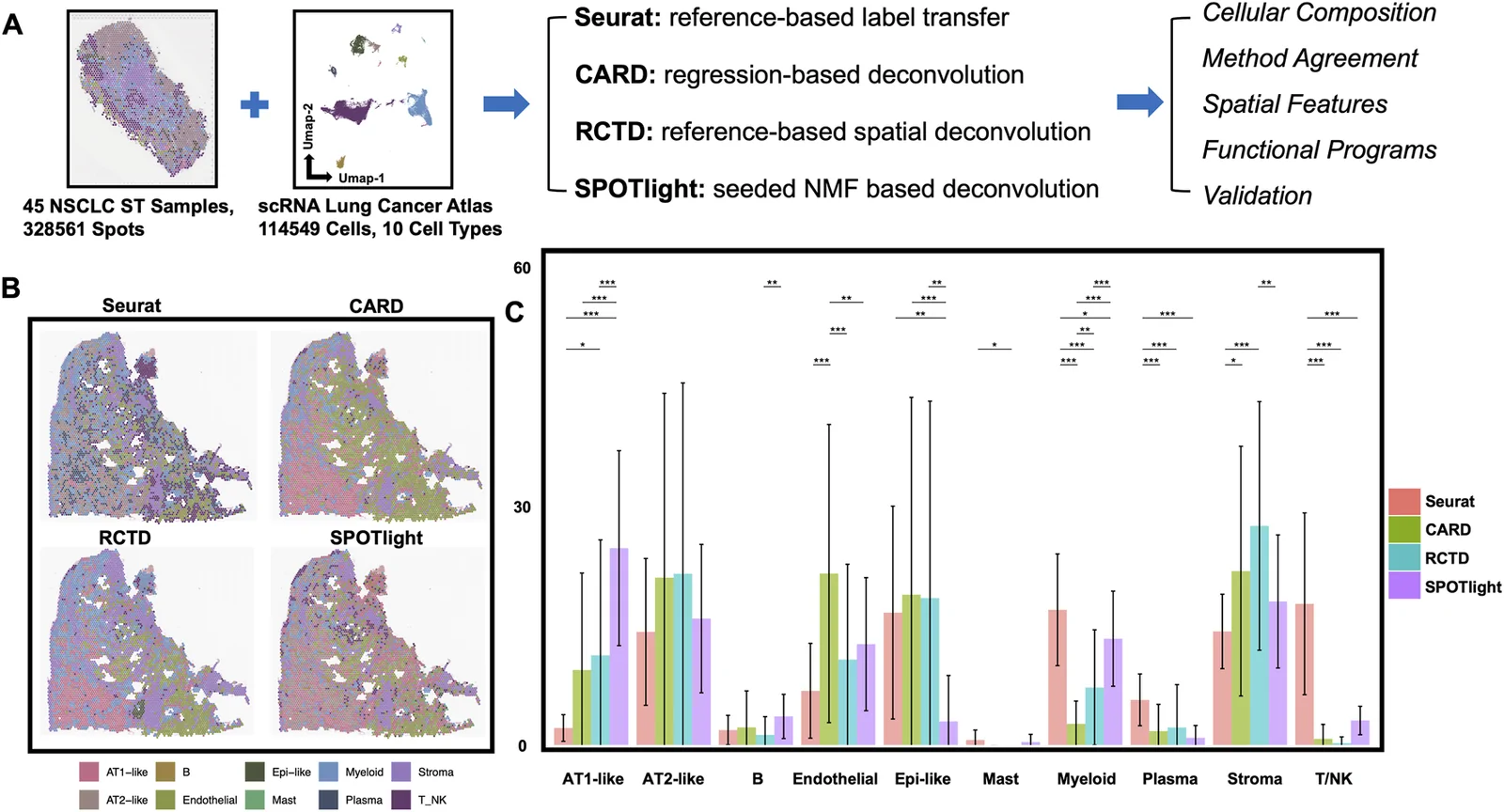
Comparative assessment of four cell-type annotation methods on NSCLC spatial transcriptomics data. **(A)** Schematic workflow of the study. A total of 45 NSCLC tumor-site Visium samples were collected from two public datasets, and 114,549 single cells from the Human Cell Atlas (10 cell types) were used as reference. Four annotation methods: Seurat (RPCA-based label transfer), CARD, RCTD, and SPOTlight were applied to the spatial transcriptomics data, resulting in 328,561 annotated spots. **(B)** Spatial visualization of cell-type distribution in a representative NSCLC sample (P1_LUAD) as predicted by each of the four methods. Each color represents a distinct cell type. **(C)** Bar plot showing the percentage of each cell type predicted by the four methods across all 45 NSCLC samples. Cell types are shown on the x-axis, and the y-axis represents the percentage. Statistical comparisons between methods were performed using pairwise Wilcoxon tests with Bonferroni correction for multiple comparisons. Only significant differences (adjusted p < 0.05) are marked: *p < 0.05, **p < 0.01, ***p < 0.001.

We selected four representative annotation methods including Seurat-based label transfer (Seurat), CARD, RCTD, and SPOTlight, based on their distinct and complementary strengths: Seurat uses RPCA to align scRNA-seq and ST data via anchor-based classification, representing the widely-used integration-based approach (Hao et al., 2024); CARD incorporates a conditional random field to model spatial dependence between neighboring spots, offering the unique advantage of leveraging spatial continuity (Ma and Zhou, 2022); RCTD fits a statistical mixture model accounting for sequencing depth and spot-level expression profiles, known for its high accuracy in distinguishing closely related cell types (Cable et al., 2022); and SPOTlight adopts a non-negative matrix factorization (NMF) regression seeded with cell-type marker genes, providing an efficient and interpretable marker-driven alternative (Elosua-Bayes et al., 2021). A total of 328,561 spots were annotated with these four methods (Figure 1A).

Using one representative example, Figure 1B shows the spatial cell-type distributions predicted by each method. Quantitative comparison of cell-type proportions across all 45 samples are illustrated in Figure 1C. Across all samples, we observed substantial method-dependent variability in estimated cell-type proportions. In particular, B cells showed remarkably similar proportions regardless of the annotation approach, suggesting that their transcriptional profiles are sufficiently distinct to be robustly identified across different computational frameworks. Endothelial cells, however, displayed clear method-specific differences, with CARD consistently predicting higher proportions compared to the other methods. This pattern may reflect the incorporation of spatial information in CARD, which can amplify signals from spatially adjacent vascular structures.

Immune populations showed notable divergence between methods. Seurat consistently predicted higher proportions of T/NK cells and myeloid cells compared to CARD, RCTD, and SPOTlight. For stromal cells, CARD and RCTD produced comparable and relatively higher estimates, whereas Seurat and SPOTlight yielded lower proportions, suggesting that modeling spot-level compositional heterogeneity contributes to improved detection of these cell types. Among tumor cell subtypes, AT2-like cells were consistently detected across all four methods, indicating that this transcriptional state is stable and robust to methodological differences. In contrast, AT1-like and Epi-like cells showed greater variability. SPOTlight detected a higher proportion of AT1-like cells but fewer Epi-like cells relative to the other methods.

### Agreement analysis uncovers cell-type-specific and method-dependent inconsistencies

**FIGURE 2:**
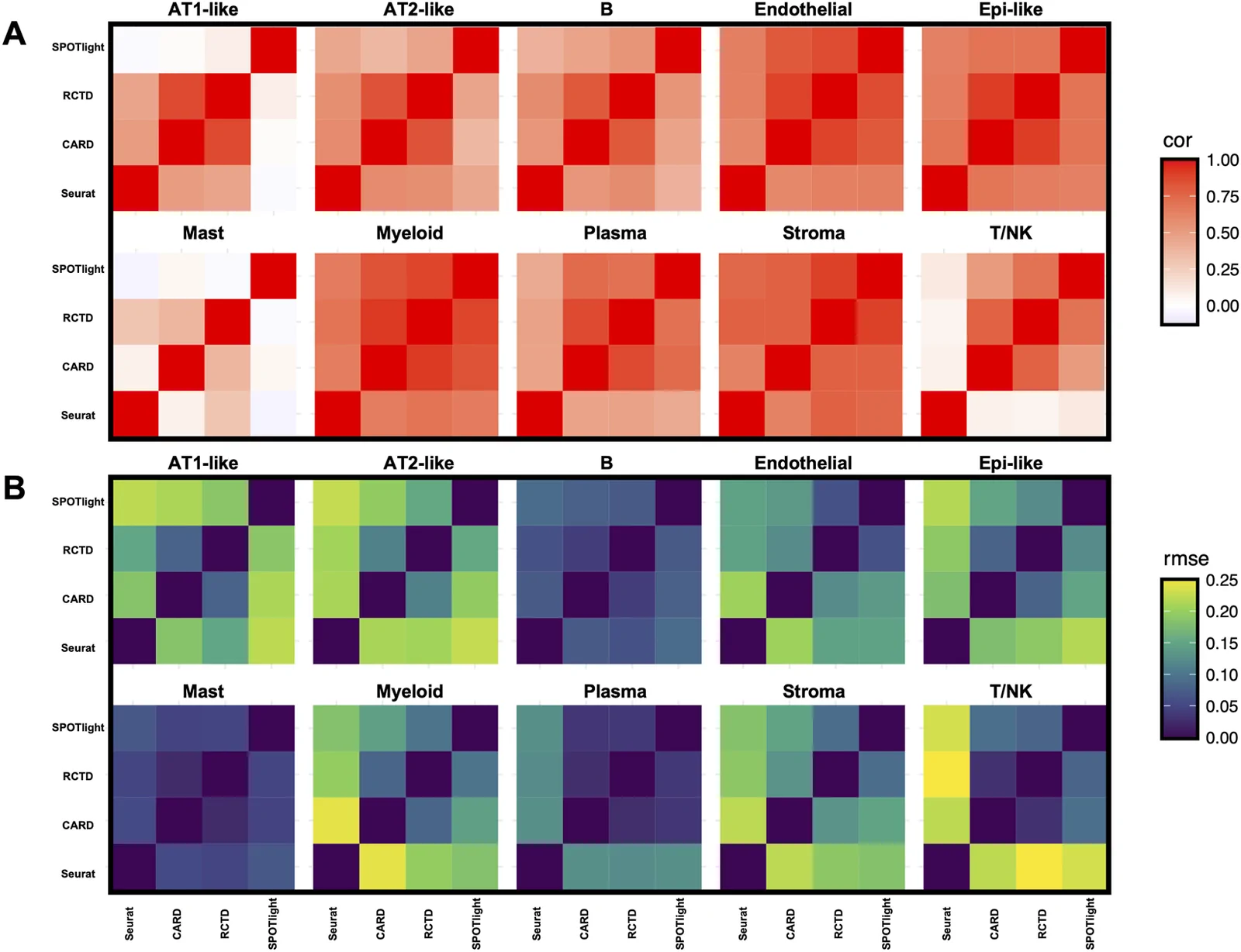
Correlation and RMSE analyses of method agreement across cell types. **(A)** Heatmap showing pairwise Pearson correlation coefficients between the four methods (Seurat, CARD, RCTD, SPOTlight) for each cell type. Color scale ranges from white (correlation = 0) to red (correlation = 1). **(B)** Heatmap showing pairwise Root Mean Square Error (RMSE) between the four methods for each cell type. Color scale from dark purple (low RMSE, high agreement) to yellow (high RMSE, low agreement).

To quantitatively assess the agreement among the four annotation methods, we performed pairwise correlation analysis across all 45 NSCLC samples for each cell type (Figure 2A). Overall, the methods exhibited moderate to high correlations for most cell types, indicating a baseline level of consistency in capturing relative abundance patterns. However, clear cell-type-specific discrepancies were observed.

SPOTlight showed substantially lower correlations with the other methods for AT1-like cells and mast cells, suggesting reduced agreement in ranking these populations across spatial locations. In contrast, Seurat displayed marked divergence specifically for T/NK cells, while CARD and RCTD consistently demonstrated high correlations with each other across all cell types, indicating strong concordance between these two approaches.

To further evaluate absolute differences in predicted proportions, we performed root mean square error (RMSE) analysis (Figure 2B). Consistent with the correlation results, AT1-like and T/NK cells showed the largest discrepancies across methods. In addition, several other cell types, including AT2-like, Epi-like, stromal, and myeloid cells, exhibited notable RMSE differences despite relatively high correlations, indicating that methods may agree on relative ranking while differing substantially in absolute abundance estimates.

Interestingly, mast cells displayed a distinct pattern characterized by low correlation but low RMSE across all method pairs. This dissociation suggests that although methods differ in the spatial ranking of mast cell abundance, their predicted proportions remain uniformly low, resulting in small absolute differences.

Across both metrics, CARD and RCTD consistently showed the highest level of agreement, with high correlation and low RMSE across all cell types. Together, these results highlight that inter-method consistency is strongly dependent on both cell type and evaluation method, and that combining complementary measures is essential for a comprehensive assessment of annotation performance.

### Spatial analysis reveals structured inter-method disagreement and its impact on spatial organization

**FIGURE 3:**
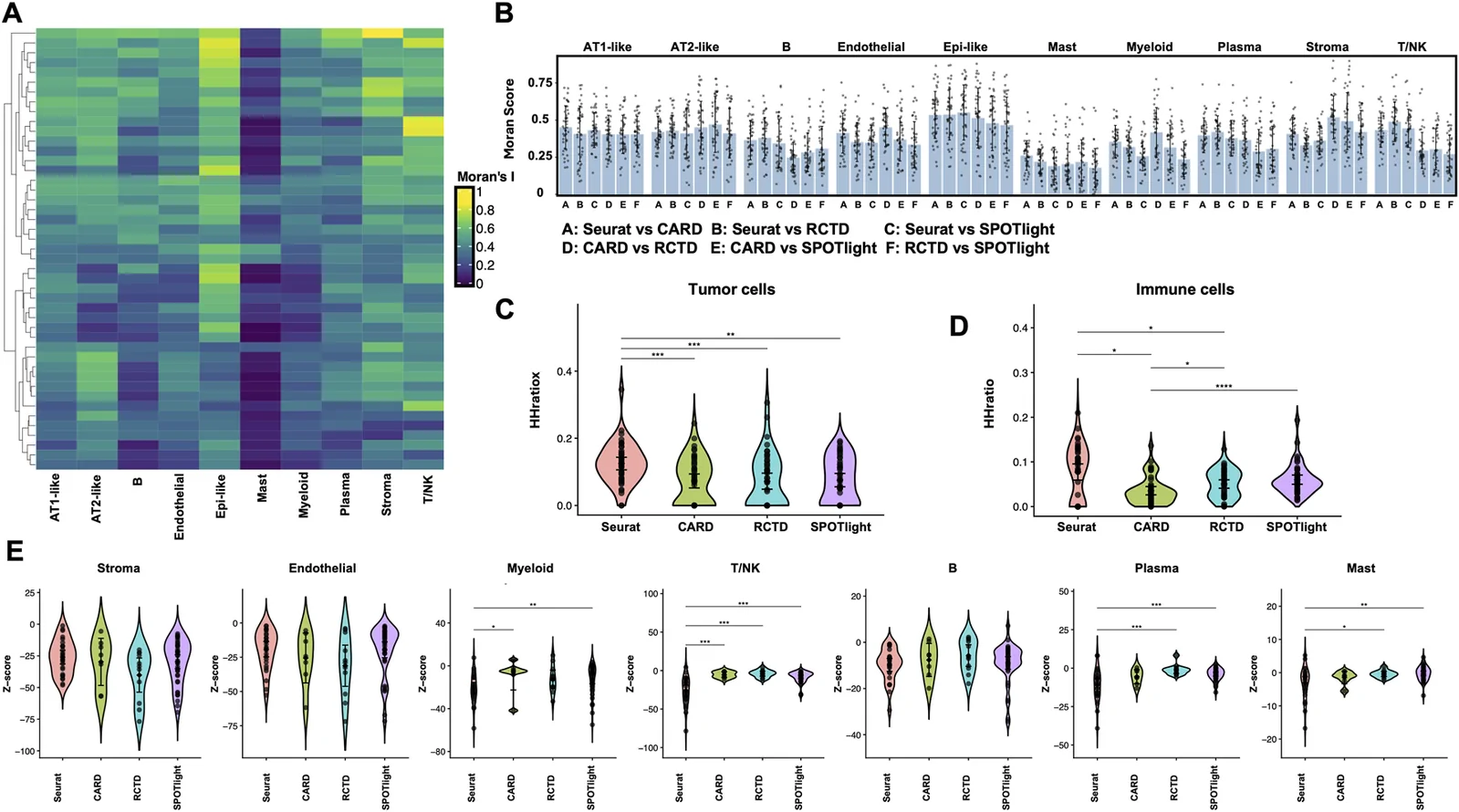
Spatial autocorrelation analysis of method agreement reveals cell-type-specific patterns and pairwise method similarity. **(A)** Heatmap showing Moran’s I values for the disagreement variance across four methods for each cell type, calculated across all 45 NSCLC samples. Color scale from dark purple (low Moran’s I) to yellow (high Moran’s I). Rows represent individual samples; columns represent cell types. **(B)** Bar plot showing mean Moran’s I values (with standard deviation as error bars) for each method pair, stratified by cell type. Each colored dot represents an individual sample’s pairwise Moran’s I value (jittered for visibility). **(C)** Violin plot showing the distribution of High-High (HH) cluster proportions for tumor cells (AT1-like, AT2-like, Epi-like) across all samples, stratified by annotation method. Each point represents an individual sample; error bars show 95% confidence intervals. Statistical comparisons were performed using pairwise Wilcoxon tests with significance levels indicated: *p < 0.05, **p < 0.01, ***p < 0.001. **(D)** Violin plot showing the distribution of High-High (HH) cluster proportions for immune cells (B, T/NK, Mast, Myeloid, Plasma) across all samples, stratified by annotation method. Same format and statistical approach as in **(C)**. **(E)** Violin plots showing the Z-scores of neighborhood enrichment between tumor and other cells across four methods. Each point represents an individual sample; bars show 95% confidence intervals. Statistical comparisons (pairwise Wilcoxon test): *p < 0.05, **p < 0.01, ***p < 0.001.

Beyond global agreement metrics, we next investigated whether inter-method discrepancies exhibit spatial structure. To determine whether discrepancies among annotation methods arise randomly or reflect underlying spatial organization, we performed Moran’s I analysis on prediction variance across methods for each cell type (Figure 3A). We found that disagreement was not randomly distributed but instead exhibited clear spatial structure. Epi-like, T/NK, and stromal cells showed relatively high Moran’s I values across samples, indicating that method disagreement is spatially clustered and often localized to specific tissue regions. In contrast, B cells, mast cells, and myeloid cells displayed consistently low Moran’s I values, suggesting either uniform agreement or uniformly low abundance across space.

Pairwise Moran’s I analysis further revealed method-specific spatial concordance (Figure 3B). For tumor cell subtypes (AT1-like, AT2-like, and Epi-like), all method pairs exhibited relatively high spatial autocorrelation, indicating that inter-method differences follow common spatial patterns shaped by tumor architecture. In contrast, mast cells showed low spatial concordance across all method combinations, consistent with their overall instability across analyses. Notably, CARD and RCTD displayed highly similar spatial patterns for stromal and myeloid cells, with only minor but spatially structured differences, reinforcing their strong agreement observed in previous analyses.

We next examined whether these spatially structured differences translate into biologically meaningful spatial organization using Local Indicators of Spatial Association (LISA) analysis (Figures 3C,D). For tumor cells, Seurat consistently identified a higher proportion of high-high (HH) clusters compared to CARD, RCTD, and SPOTlight, which showed comparable clustering levels. A similar trend was observed for immune cells, although Seurat exhibited greater variability across samples.

Finally, we assessed spatial cell-cell interactions using neighborhood enrichment analysis (Figure 3E). While tumor-stroma, tumor-endothelial, and tumor-B cell interactions were consistently detected across all methods, substantial divergence was observed for several tumor-immune pairs. Seurat showed markedly stronger spatial repulsion between tumor cells and T/NK, myeloid, plasma, and mast cells, whereas the other methods displayed more moderate or near-neutral interaction patterns.

Together, these results demonstrate that inter-method discrepancies are spatially structured and can substantially influence the interpretation of spatial clustering and cell-cell interactions in the tumor microenvironment.

### Functional evaluation reveals method-specific strengths in capturing cell identity and pathway activity

**FIGURE 4:**
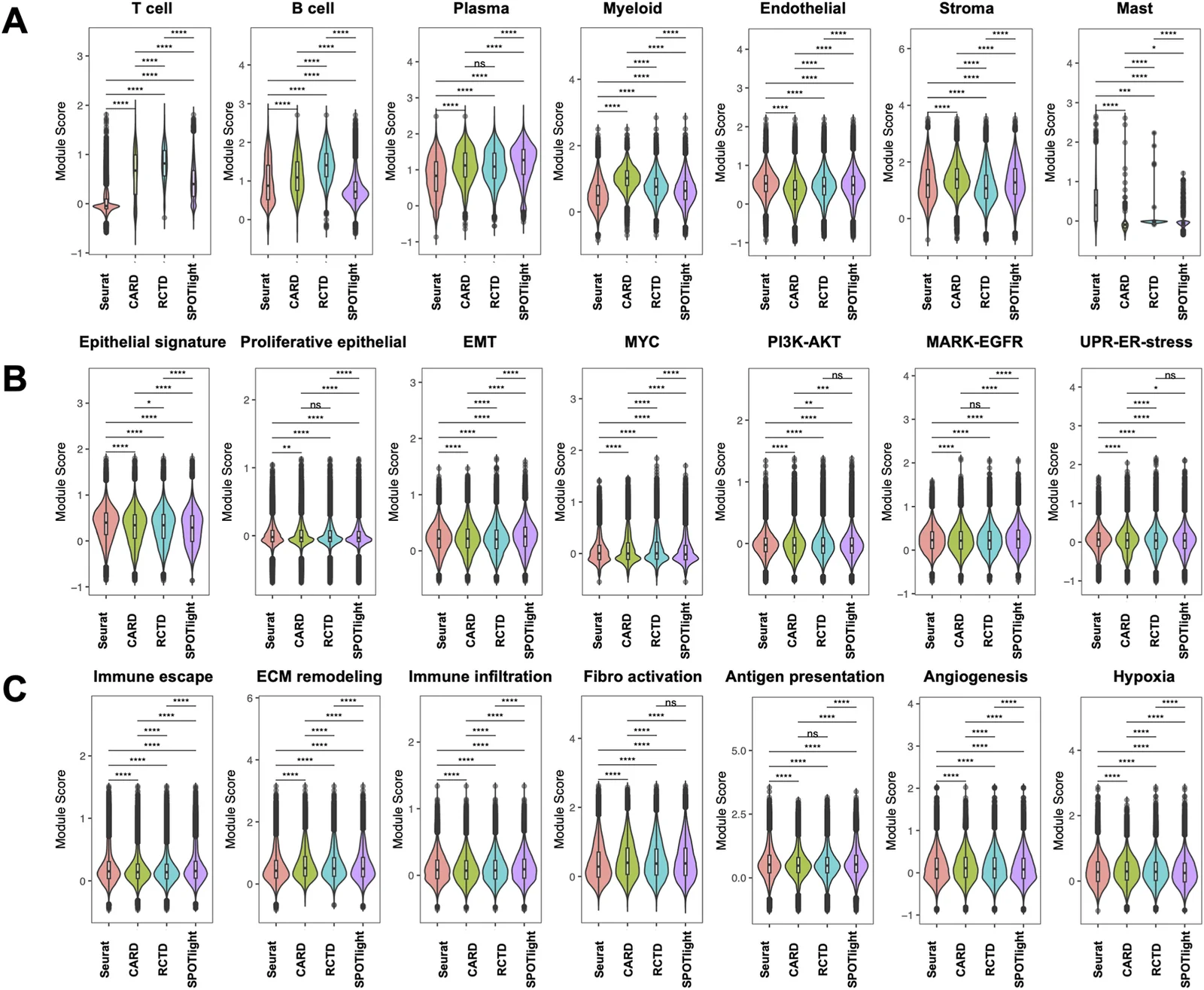
Module score-based assessment of method performance. **(A)** Violin plots of canonical marker module scores for seven TME cell types by method. Points represent individual spots; boxplots show median and IQR. Pairwise Wilcoxon test: *p < 0.05, **p < 0.01, ***p < 0.001, ****p < 0.0001. **(B)** Violin plots of seven tumor-related pathway module scores for tumor cells. Same format as **(A)**. **(C)** Violin plots of seven TME functional pathway module scores for TME cells. Same format as **(A)**.

To further evaluate the biological implications of method-specific differences, we quantified module scores based on canonical marker genes for major tumor microenvironment (TME) cell types (Figure 4A) (Yang et al., 2024; Travaglini et al., 2020). Distinct method-specific performance patterns were observed across cell lineages. CARD and RCTD consistently achieved the highest module scores for T cells, myeloid cells, and stromal cells, suggesting improved accuracy in capturing canonical transcriptional identities for these populations. In contrast, SPOTlight showed the highest scores for B cells, consistent with its marker-driven framework. Endothelial cells exhibited comparable scores across all methods, indicating that this cell type is robustly identified regardless of annotation strategy. Notably, mast cells showed substantial variability across methods, reflecting the inherent difficulty of accurately identifying this sparse and transcriptionally overlapping population.

We next evaluated functional pathway activity in tumor cells using curated cancer-related gene sets (Figure 4B) (Tirosh et al., 2016; Liberzon et al., 2015; Liberzon et al., 2011). CARD and RCTD showed highly consistent module scores across pathways, including proliferative epithelial and MAPK-EGFR signaling, reinforcing their strong agreement observed in previous analyses. In contrast, Seurat diverged significantly from the other methods across all pathways, indicating that it identifies a distinct subset of tumor-associated transcriptional programs. Despite differences in tumor cell abundance, pathway-level scores were broadly comparable across methods, suggesting convergence in functional characterization of tumor cells.

In the TME, functional pathway analysis revealed a different pattern (Figure 4C) (Liberzon et al., 2015; Subramanian et al., 2005). CARD consistently produced higher scores for stromal activation pathways, including extracellular matrix remodeling, fibroblast activation, and angiogenesis, whereas SPOTlight showed stronger signals for immune-related pathways such as immune infiltration and immune escape. Seurat exhibited intermediate pathway scores but greater variability across samples. Interestingly, CARD and RCTD, despite their high agreement in cell-type composition, showed divergence across several TME functional pathways, indicating that similar cell-type assignments do not necessarily imply concordant functional inference.

Overall, these results demonstrate that no single method universally outperforms others; instead, method performance is context-dependent and varies across cell types and biological pathways.

### Cross-method consensus spatial programs and independent scRNA-seq validation reveal robust spatial gene expression programs in NSCLC

**FIGURE 5:**
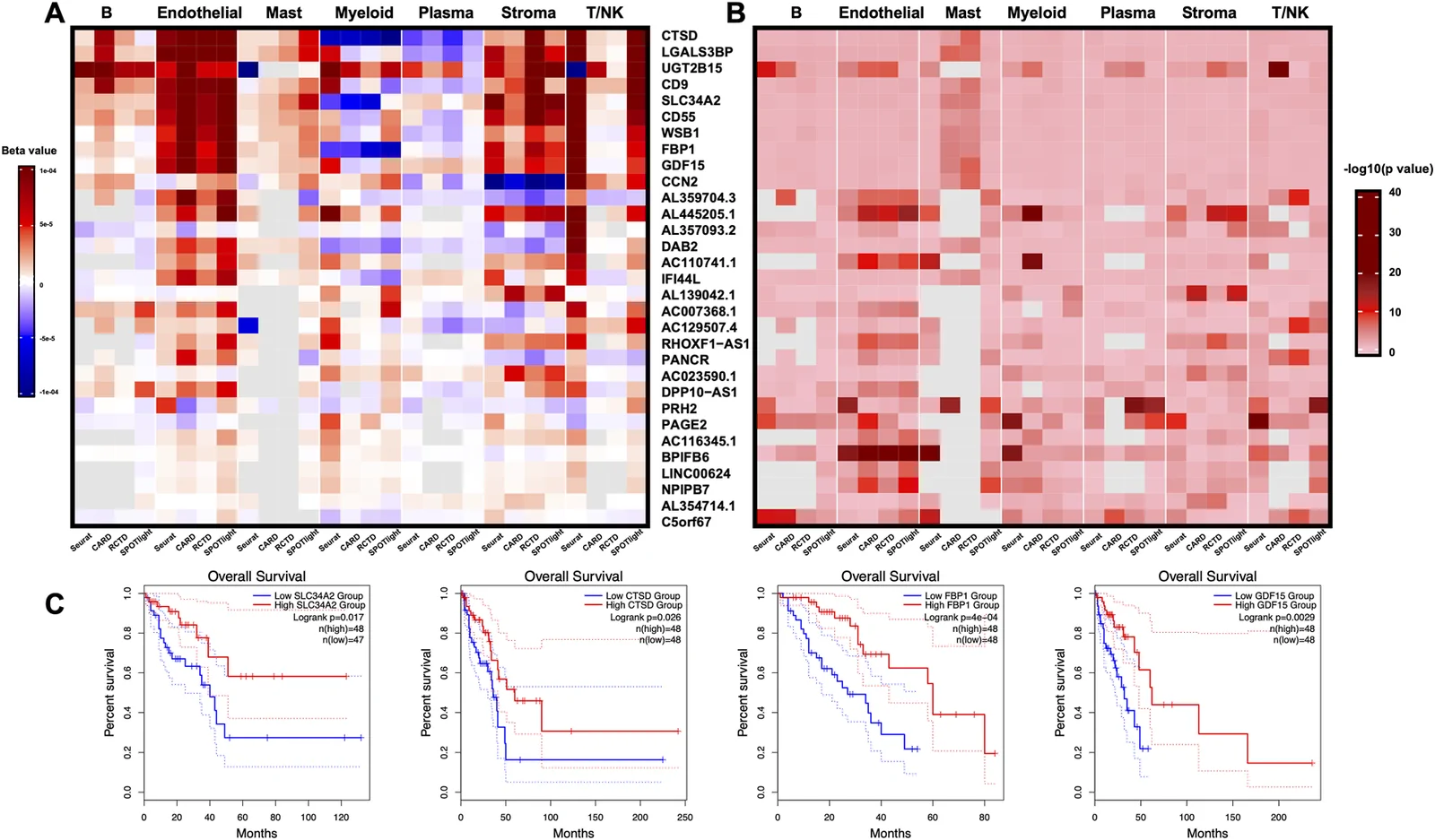
Cross-method consensus reveals biologically relevant gene expression gradients in the NSCLC microenvironment. **(A)** Heatmap showing beta coefficients (gradient of gene expression with respect to distance to tumor cells) across methods. Red indicates positive beta (increasing expression with distance), blue indicates negative beta (decreasing expression with distance). Columns are grouped by cell type, with four methods shown per cell type. Row names indicate genes showing consensus across methods. **(B)** Heatmap showing -log10 (p-values) corresponding to the beta coefficients in **(A)**, serving as statistical significance validation. Darker red indicates higher statistical significance (smaller p-values). Columns are grouped by cell type, with four methods shown per cell type. Row names indicate genes showing consensus across methods. **(C)** Kaplan-Meier survival curves for CTSD, SLC34A2, FBP1, and GDF15 in lung adenocarcinoma patients from the GEPIA2 database (90% vs. 10% expression cutoff). Low expression of all four genes is associated with significantly worse prognosis.

To identify biologically robust signals independent of annotation strategy, we focused on genes exhibiting consistent spatial expression gradients across all four methods (Figures 5A,B). By requiring concordance and significance in at least two methods, we aimed to extract gene-level patterns that are resilient to methodological variability and therefore more likely to reflect true spatial biology in the tumor microenvironment.

Among all cell types, endothelial and stromal compartments showed the highest degree of cross-method concordance. A substantial number of genes consistently displayed directional spatial gradients relative to these compartments, indicating stable transcriptional programs associated with tissue architecture. In particular, genes such as CTSD, LGALS3BP, UGT2B15, CD9, SLC34A2, CD55, WSB1, FBP1, and GDF15 showed consistent positive spatial gradients across methods, suggesting increased expression with distance from endothelial and stromal regions. These conserved patterns likely reflect coordinated regulation of tumor-stroma and tumor-vasculature interactions.

In contrast, immune-associated compartments exhibited more heterogeneous spatial patterns, with weaker cross-method agreement. Genes such as CTSD, SLC34A2, and FBP1 showed negative spatial gradients in myeloid-associated contexts, whereas CD9 and CCN2 exhibited positive gradients in T/NK-associated regions. This suggests that spatial transcriptional programs in immune compartments are more context-dependent and potentially influenced by local microenvironmental heterogeneity.

Among these consensus genes, we selected SLC34A2, FBP1, CTSD, and GDF15 for survival exploration based on their robust cross-method concordance and previously documented roles in lung cancer biology. For example, SLC34A2 has been reported as a tumor suppressor involved in regulating lung adenocarcinoma progression (Zhu et al., 2025), while FBP1 suppresses tumor stemness through metabolic and ubiquitination-related mechanisms (He et al., 2024). CTSD and GDF15 have also been implicated in tumor progression and therapy resistance, further supporting the biological relevance of these spatially consistent signals (Melero et al., 2025; Liu et al., 2020).

Finally, we performed an exploratory survival analysis using the GEPIA2 database and observed that lower expression of SLC34A2, CTSD, FBP1, and GDF15 showed a trend toward worse overall survival (Figure 5C). These preliminary observations, together with their consistent spatial gradients and known functional roles, suggest that cross-method consensus may help identify genes with potential biological and clinical relevance warranting further investigation.

**FIGURE 6:**
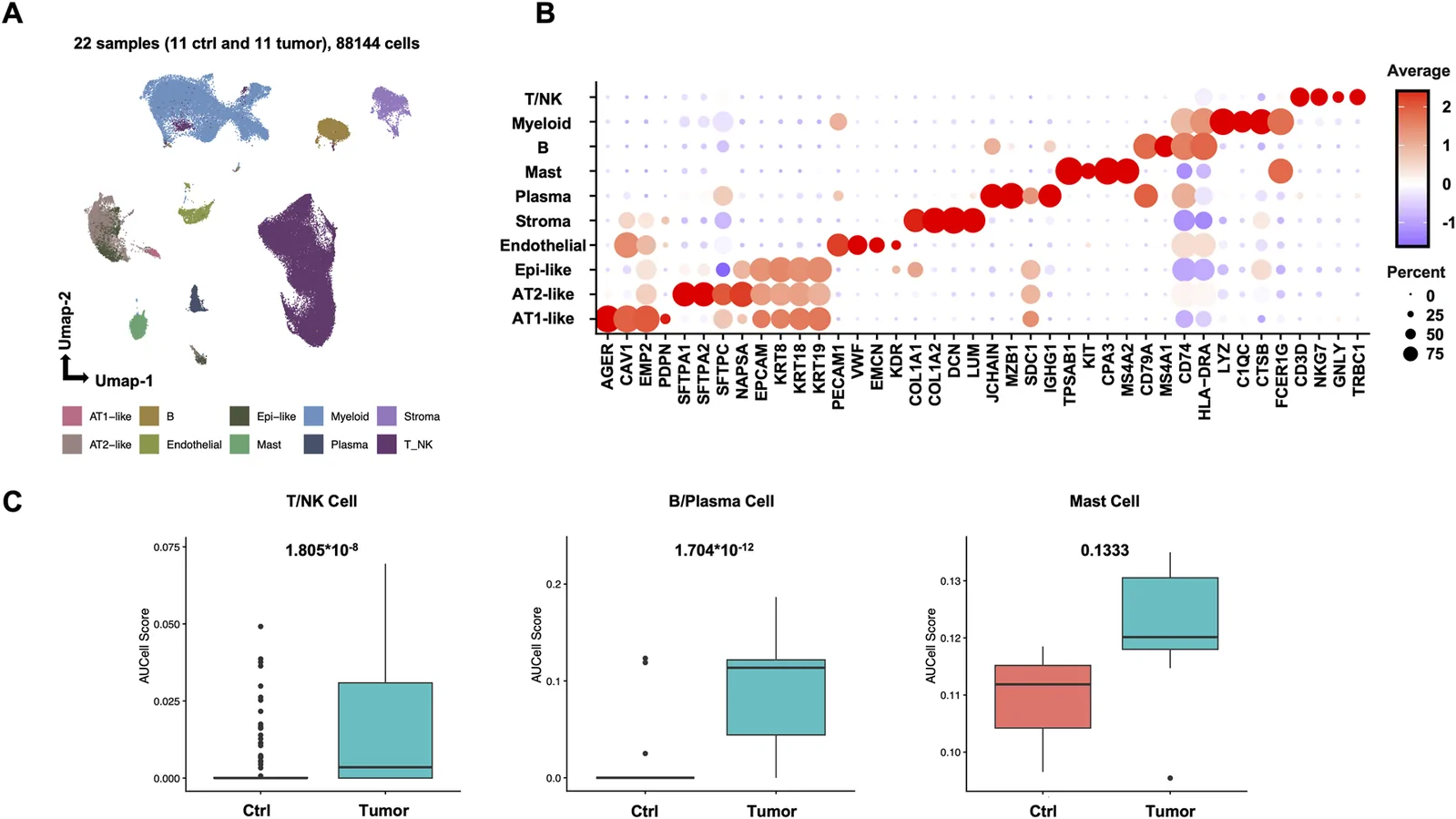
Independent single-cell validation of cross-method consensus spatial markers in NSCLC. **(A)** UMAP visualization of the independent single-cell RNA sequencing cohort (11 control and 11 tumor samples; >88,000 cells). **(B)** Dot plot showing the expression patterns of consensus marker genes across major annotated cell populations, confirming consistent cell-type specificity. **(C)** Boxplot comparison of consensus gene set module scores in T/NK, B/PC, and myeloid compartments between control and tumor samples. Each boxplot displays median, interquartile range, and individual outlier values. Exact P values are shown above each comparison.

To further validate the biological robustness of the cross-method consensus markers identified from spatial transcriptomic analyses, we examined their expression patterns in an independent single-cell RNA sequencing cohort comprising 11 control lung samples and 11 tumor samples, totaling over 88,000 cells (GSE131907) (Kim et al., 2020). Using the same marker genes, we performed cell-type annotation in this independent dataset, successfully identifying the same 10 major populations. UMAP visualization demonstrated clear separation of annotated cell populations, while dot plot analysis confirmed the conserved expression of these marker genes across corresponding cell types (Figures 6A,B).

Next, we aggregated the previously identified consensus spatial gradient genes into cell-type-specific gene sets and performed module scoring within T/NK cells, B/Plasma cells (B/PCs), and myeloid cells. Consistent with our spatial transcriptomic findings, tumor-derived T/NK and B/PCs exhibited significantly elevated consensus gene set scores compared with control samples (P < 0.05; Figure 6C), supporting the reproducibility of these transcriptional programs across independent datasets and modalities. Myeloid cells also demonstrated increased consensus scores in tumor samples, although this trend did not reach statistical significance (P = 0.1333), suggesting greater heterogeneity or context dependency within myeloid-associated transcriptional programs.

### Cross-method consensus transcription factor programs and scRNA-seq validation reveal conserved regulatory architecture in NSCLC

**FIGURE 7:**
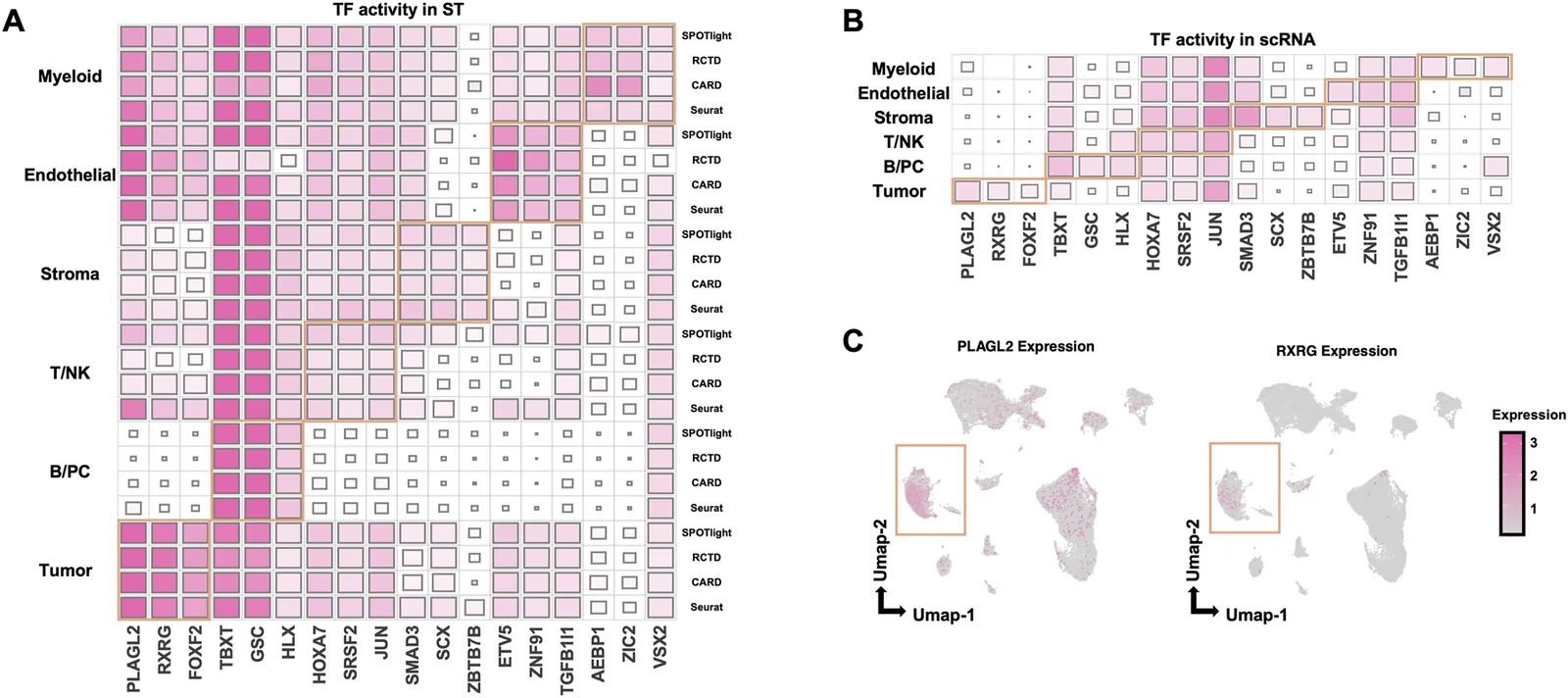
Cross-method and cross-platform validation of conserved transcription factor activity programs in NSCLC. **(A)** Heatmap summarizing the top active transcription factors inferred by decoupleR for each cell type across four spatial annotation methods, including tumor, B/PC, T/NK, stromal, endothelial, and myeloid compartments. **(B)** Validation of consensus transcription factor activity in an independent single-cell RNA sequencing cohort. TF activity scores for selected consensus regulators were assessed across corresponding cell populations. **(C)** UMAP visualization of PLAGL2 and RXRG expression in tumor samples from the independent single-cell RNA sequencing dataset. Color intensity indicates relative gene expression levels, with darker colors representing higher expression.

To further extend gene-level spatial consensus signals into upstream regulatory mechanisms, we inferred transcription factor (TF) activity across tumor cells and major TME cells using decoupleR-based regulatory network analysis with four spatial annotation methods. For each cell type, TFs that were consistently identified as highly active across all four spatial annotation methods were selected to represent dominant regulatory programs (Figure 7A).

This comparative framework enabled identification of robust TF activity patterns that were reproducible despite methodological variability. We further validated these consensus TF programs in the independent single-cell RNA sequencing tumor samples by assessing TF activity across corresponding cell types (Figure 7B). In tumor cells, PLAGL2 and RXRG showed robust activation, consistent with their reported roles in tumor progression, stemness maintenance, and transcriptional reprogramming (Joseph et al., 2019; Yang et al., 2025). Stromal and endothelial compartments demonstrated strong enrichment of SMAD3 and TGFB1I1, suggesting activation of TGF-β-associated remodeling pathways that may contribute to extracellular matrix reorganization, angiogenesis, and tumor-stroma communication (Luo, 2017). In immune-related compartments, JUN and HLX exhibited elevated activity, potentially reflecting adaptive immune signaling and context-dependent differentiation states (Becknell et al., 2007; Scopa et al., 2023). Notably, developmental regulators such as TBXT and GSC were also recurrently activated, implying that lineage plasticity and embryonic transcriptional programs may contribute to malignant progression and microenvironmental adaptation (Daneshvar et al., 2016; Wang et al., 2025).

In addition, we further examined the expression of PLAGL2 and RXRG in tumor-derived cells from this independent single-cell dataset and confirmed that both genes were consistently highly expressed in tumor cells, further supporting their robust tumor-associated regulatory roles (Figure 7C).

### Tumor-proximity associated pathway reprogramming reveals conserved TME states across spatial methods and scRNA-seq validation

Building upon both gene-level and regulatory-level spatial consensus, we next investigate how tumor proximity shapes transcriptional pathway programs within the tumor microenvironment (TME). For each spatial sample, TME cells were stratified into tumor-proximal and tumor-distal groups based on their relative spatial distance to malignant regions. Differential expression analysis was then independently performed across all four annotation frameworks (Seurat, CARD, RCTD, and SPOTlight), followed by Gene Set Enrichment Analysis (GSEA) to identify biological pathways associated with tumor proximity.

**FIGURE 8:**
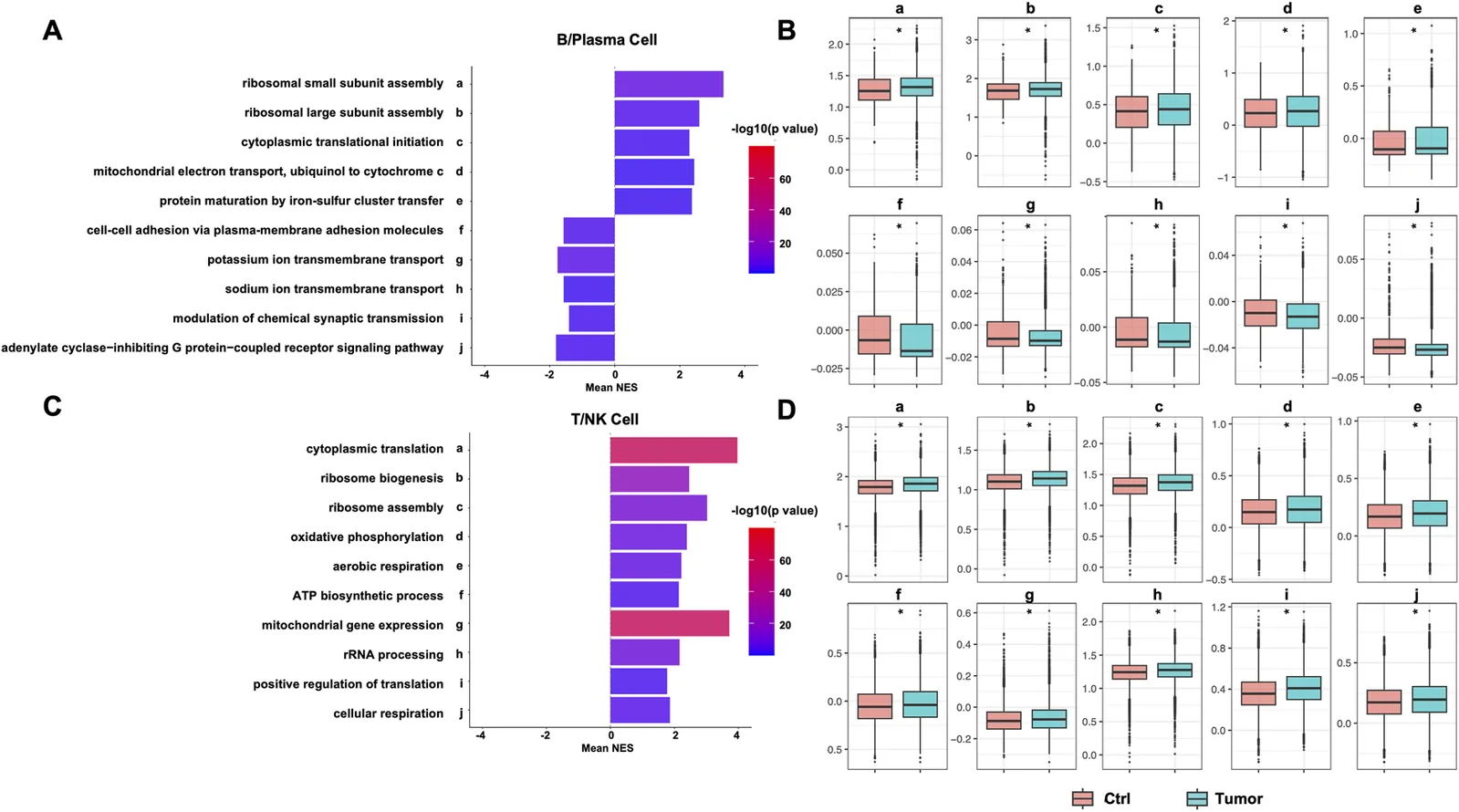
Conserved tumor-associated pathway reprogramming in B/PCs and T/NK cells across spatial annotation methods and independent single-cell validation. **(A,C)** Gene Set Enrichment Analysis (GSEA) of tumor-proximal versus tumor-distal B/PCs and T/NK cells across four annotation methods. The x-axis represents normalized enrichment score (NES), indicating pathway activation or suppression, while color intensity (red to blue) reflects the magnitude of statistical significance as -log10 (p value). Representative biologically relevant pathways are labeled with corresponding letter annotations a-j. **(B,D)** Boxplot-based validation of corresponding pathway activity scores in independent single-cell RNA-seq datasets comparing tumor-derived (Tumor) and normal-derived (Ctrl) B/PCs and T/NK cells. Pathway labels directly correspond to annotated pathways (a-j) shown in panels **(A,C)**. Statistically significant differences (p < 0.05) are indicated by asterisks (*).

**FIGURE 9:**
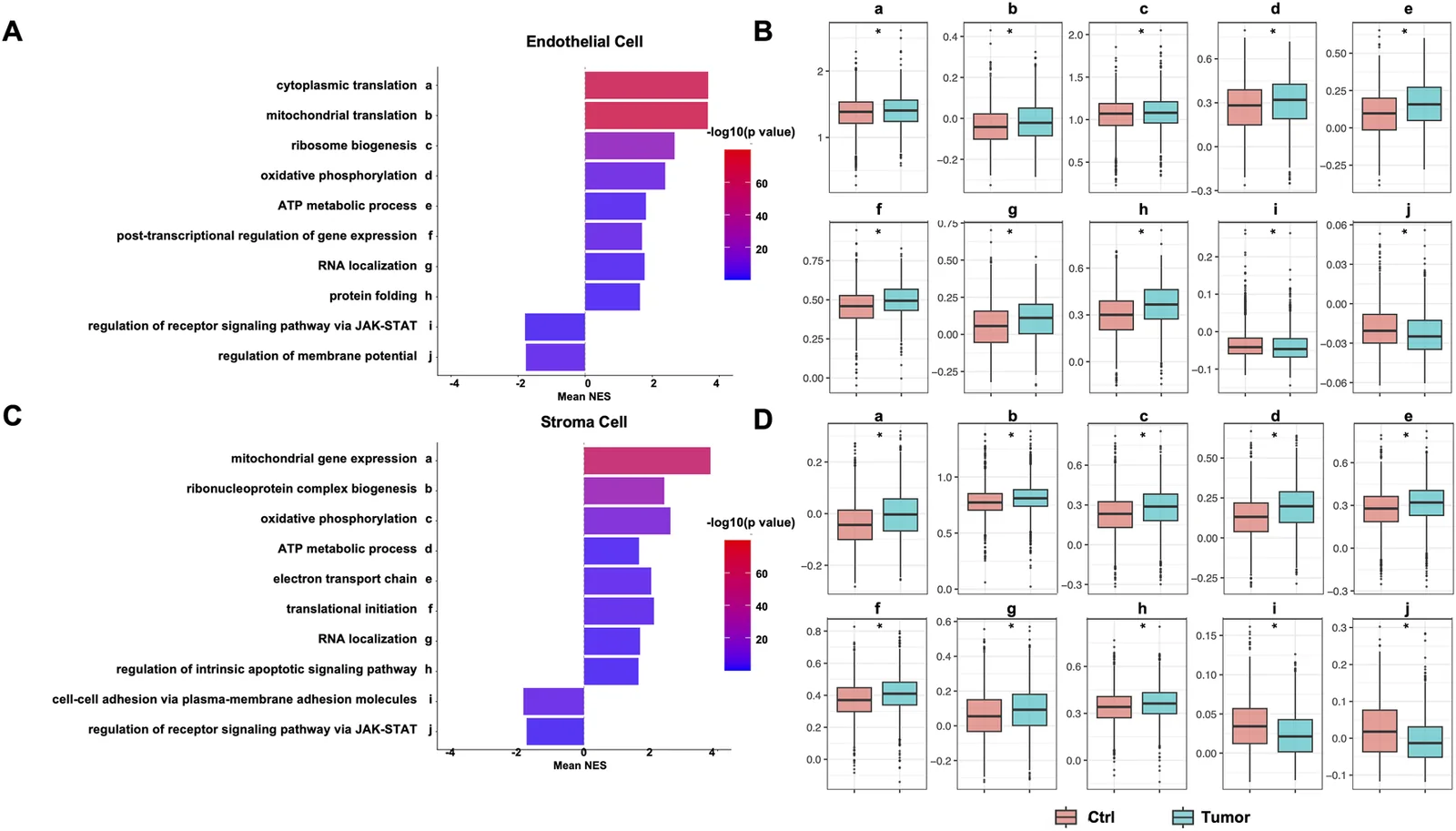
Conserved tumor-associated pathway reprogramming in endothelial and stroma cells across spatial annotation methods and independent single-cell validation. **(A,C)** Gene Set Enrichment Analysis (GSEA) of tumor-proximal versus tumor-distal endothelial and stroma cells across four annotation methods. The x-axis represents normalized enrichment score (NES), indicating pathway activation or suppression, while color intensity (red to blue) reflects the magnitude of statistical significance as -log10 (p value). Representative biologically relevant pathways are labeled with corresponding letter annotations a-j. **(B,D)** Boxplot-based validation of corresponding pathway activity scores in independent single-cell RNA-seq datasets comparing tumor-derived (Tumor) and normal-derived (Ctrl) endothelial and stroma cells. Pathway labels directly correspond to annotated pathways (a-j) shown in panels **(A,C)**. Statistically significant differences (p < 0.05) are indicated by asterisks (*).

By intersecting pathway-level enrichment results across all four annotation methods, we identified robustly conserved transcriptional programs for each cell type, revealing both shared activation and suppression patterns despite methodological variability (Figures 8A,C, 9A,C). Across multiple TME compartments, tumor-associated cells consistently exhibited enhanced translational activity, ribosome biogenesis, and oxidative phosphorylation, suggesting widespread metabolic and biosynthetic reprogramming in response to the tumor niche. In contrast, pathways related to intercellular signaling, membrane transport, adhesion, and immune-associated signaling were recurrently suppressed, indicating progressive functional specialization or dysregulation within tumor-associated states. Notably, T/NK cells displayed particularly strong upregulation of mitochondrial respiration and protein synthesis pathways, while stromal and endothelial compartments showed broader suppression of signaling pathways such as JAK-STAT and ion transport regulation.

To further validate these conserved tumor-associated programs, we leveraged the independent single-cell RNA-seq cohort containing cells derived from tumor and normal tissue origins. For each corresponding cell type, pathway activity scores were calculated for the conserved signatures identified from spatial analyses, and comparisons between tumor-derived and normal-derived cells demonstrated significant recapitulation of the same biological programs (Figures 8B,D, 9B,D). This orthogonal validation confirmed that the transcriptional remodeling observed in spatially tumor-proximal cells reflects broader tumor-associated cellular state transitions rather than platform-specific artifacts, reinforcing the biological robustness of these conserved TME alterations.

## Discussion

Our systematic evaluation of four widely used spatial transcriptomics annotation frameworks in NSCLC reveals that computational strategy significantly influences the interpretation of tumor microenvironment (TME) organization. While all methods capture broad cellular structure, we observe substantial variability in inferred cell-type composition, spatial patterns, and functional pathway activity, particularly within immune, stromal, and epithelial compartments. These discrepancies highlight that spatial inference is inherently method-sensitive, especially in regions of high cellular heterogeneity such as tumor-stroma interfaces.

These method-dependent discrepancies can be partially explained by the distinct algorithmic principles underlying each approach. Seurat’s anchor-based transfer learning prioritizes global transcriptomic alignment, which may systematically over-project abundant immune populations from the scRNA-seq reference, accounting for its consistently higher T/NK and myeloid cell estimates (Stuart et al., 2019). In contrast, CARD incorporates spatial continuity via conditional random fields, which tends to amplify signals from spatially contiguous structures such as vascular networks and fibrotic niches, explaining its elevated endothelial and stromal proportions (Ma and Zhou, 2022). RCTD’s statistical mixture model, by explicitly modeling spot-level compositional heterogeneity, achieves high resolution for cell types with distinct molecular profiles, contributing to its strong agreement with CARD for stromal cells (Cable et al., 2022). SPOTlight’s marker-seeded NMF regression, while efficient and interpretable, is more sensitive to marker gene quality and overlap, particularly for populations with less distinctive transcriptional signatures such as mast cells and AT1-like cells, leading to its reduced concordance with other methods for these lineages (Elosua-Bayes et al., 2021). Thus, the observed variability reflects not merely technical noise but the inherent biases introduced by each algorithm’s core assumptions.

Despite these differences at the compositional level, we identify a set of transcriptional signals that are consistently recovered across methods, representing computationally robust observations that require further experimental validation. Spatially conserved programs emerge, including genes such as SLC34A2, FBP1, and CTSD, as well as transcriptional regulators including PLAGL2, RXRG, and SMAD3. These signals define coherent spatial gradients and regulatory states that are largely independent of annotation strategy, suggesting the presence of a stable underlying transcriptional architecture within the TME. Functionally, these consistently observed programs converge on metabolic and biosynthetic processes, including translation, oxidative phosphorylation, and ribosome biogenesis, while immune- and signaling-related pathways are broadly suppressed in tumor-proximal regions (Arner and Rathmell, 2023; Wegiel et al., 2018). This spatial suppression of immune-associated programs, coupled with the activation of metabolic pathways, provides a potential mechanistic framework for understanding how tumor proximity may contribute to immune dysfunction within the TME, consistent with the known metabolic reprogramming that accompanies immune cell adaptation in tumors (Zheng et al., 2024).

Importantly, these spatially derived programs are supported by independent single-cell RNA-sequencing data, where tumor-derived cells recapitulate the same gene signatures and pathway activities observed in spatial analyses. This cross-modal consistency indicates that tumor proximity-associated transcriptional changes reflect genuine biological state transitions rather than method-specific artifacts. However, we acknowledge that scRNA-seq data cannot directly validate spatial gradients, neighborhood relationships, or spatial autocorrelation patterns, as these are inherently spatial properties. The spatial findings presented here therefore remain computational predictions that await orthogonal validation using spatially resolved experimental approaches. Together, these results support a model in which spatial transcriptomic data contains a conserved biological core that is robust across computational frameworks, embedded within a layer of method-dependent quantitative variability.

A key implication of this work is that relying on a single spatial annotation method may lead to incomplete or biased biological interpretation, whereas consensus-based analysis enables the identification of reproducible spatial programs with potential clinical relevance. However, several limitations should be acknowledged. First, although we evaluated four representative deconvolution frameworks with distinct modeling assumptions, additional emerging methods may capture complementary aspects of spatial heterogeneity not covered here (Jia et al., 2025) And spatial resolution and spot-level mixing inherent to Visium data may obscure finer-scale cellular interactions, potentially attenuating cell-type-specific spatial signals (Liu et al., 2024). Crucially, a fundamental challenge remains the absence of a ground truth for the annotations. While our multi-method comparison reveals consensus patterns, these should not be equated with biological verity (Li et al., 2023; Li et al., 2022).

Although this study focused on NSCLC ST data, several considerations are relevant for broader application, such as the need for multi-method comparison and consensus-based interpretation, may be applicable to other solid tumors as many cancers share spatial features including tumor-stroma interfaces, immune infiltration, and metabolic gradients (Kang et al., 2025). However, direct extrapolation should be cautious, as tumor-specific tissue architecture, stromal composition, immune contexture, and malignant cell states may influence both spatial signals and algorithm performance (Fu et al., 2021; Ma et al., 2026). In addition, annotation methods may show different compatibility across spatial platforms due to differences in resolution, gene coverage, capture efficiency, and cell segmentation strategy (Ding et al., 2025; Lim et al., 2025). Therefore, future studies integrating higher-resolution spatial technologies, non-Visium NSCLC datasets, and additional solid tumor type will be important for evaluating the generalizability and robustness of spatial annotation frameworks, as well as for refining the mechanistic understanding of these conserved tumor-microenvironment programs.

## Methods

### Spatial transcriptomics data collection and processing

A total of 45 NSCLC tumor-site Visium samples were collected from two public datasets. Specifically, 20 samples were derived from Spatial transcriptomics (10x Visium) of human NSCLC lesions (De Zuani et al., 2024) and 25 samples were obtained from GSE307534, selecting samples annotated as tumor site. For data processing, samples from different sources were loaded using the Load10X_Spatial function from Seurat (v5) (Yang et al., 2024). All samples were merged into a single Seurat object containing spatial coordinates and expression counts, with sample identity preserved for downstream analyses. Regarding batch effect consideration, all 45 samples were derived from tumor regions of non-small cell lung cancer, ensuring biological consistency across samples. For algorithm benchmarking, we employed a within-sample evaluation strategy where all four deconvolution methods, including Seurat label transfer, CARD, RCTD, and SPOTlight, were independently applied to each spatial sample using the same single-cell reference dataset. This design ensures that technical variations between samples, such as sequencing depth and batch effects, equally affect all methods within each sample, and algorithm performance comparisons are based on relative rankings within individual samples rather than absolute values across samples.

### Single-cell RNA-seq datasets

A comprehensive single-cell reference was constructed using all cancer-related data from the Human Cell Atlas Lung Biological Network (https://data.humancellatlas.org/hca-bio-networks/lung/atlases/lung-v1-0) (Sikkema et al., 2023). The dataset comprised 114,549 cells classified into 10 cell types. The raw count matrix was loaded and converted to a Seurat object. Cells were filtered based on mitochondrial percentage, number of features, and number of counts. The data were normalized, variable features identified and scaled. Harmony integration was performed to correct for donor-specific batch effects, followed by PCA, neighborhood graph construction, and UMAP visualization. Clusters were manually annotated into 10 cell types based on canonical marker genes. For independent validation, an additional single-cell RNA sequencing cohort comprising 22 samples (11 normal lung controls and 11 tumor samples), totaling over 88,000 cells, was analyzed from GSE131907.

### Seurat-based label transfer

For each spatial sample, the query object was normalized and variable features identified. Transfer anchors were identified between the reference scRNA-seq object and each spatial query using the FindTransferAnchors function with RPCA reduction (dims = 1:30). Cell type labels were then transferred using TransferData, and prediction scores were calculated. The predicted cell type with the highest score was assigned to each spatial spot. The resulting prediction score matrices were extracted and combined across all samples.

### CARD deconvolution

Spatial count matrices and coordinate data were extracted for each sample separately. The reference scRNA-seq counts and cell type annotations were used to construct a CARD object. Deconvolution was performed using CARD, which employs a conditional random field model to incorporate spatial dependence between neighboring spots. The resulting cell-type proportion matrices were extracted and added to the Seurat object metadata, with the dominant cell type assigned as the final annotation for each spot.

### RCTD deconvolution

A Reference object was constructed from the scRNA-seq count matrix and cell type factor. Spatial counts and coordinates were extracted for each sample and used to create RCTD objects. RCTD deconvolution was performed and the posterior probability weights were extracted from the results, and the cell type with the maximum weight was assigned to each spot. Weight matrices were combined across all samples for downstream analysis.

### SPOTlight annotation

The scRNA-seq reference was converted to a SingleCellExperiment object and normalized. Marker genes for each cell type were identified using FindAllMarkers with threshold of log2 fold change >0.5 and minimum detection percentage of 25%. The top 50 marker genes per cell type were selected based on average log2 fold change. For each spatial sample, SPOTlight was run using the NMF-based regression approach with the marker gene set and highly variable genes as input. The resulting deconvolution matrices were extracted, and the cell type with the maximum proportion was assigned to each spot.

### Cell-type composition analysis

The proportion of each cell type per sample per method was calculated. Bar plots showing mean percentage with standard deviation were generated, with individual sample proportions overlaid as points. Pairwise comparisons between methods were performed using Wilcoxon rank-sum tests with Bonferroni correction for multiple comparisons. Significance levels were annotated as *p < 0.05, **p < 0.01, ***p < 0.001.

### Correlation and RMSE analysis

For each cell type, Pearson correlation coefficients were calculated between every pair of methods based on spot-level prediction scores across all samples. RMSE (Root Mean Square Error) was computed as the square root of the mean squared difference between paired method predictions. Both metrics were visualized as heatmaps, with correlation heatmaps using a blue-white-red gradient (blue = −1, white = 0, red = 1) and RMSE heatmaps using the viridis color scale (dark purple = low RMSE/high agreement, yellow = high RMSE/low agreement). For statistical inference, correlation and RMSE values were first calculated per sample and then compared across methods using sample-level paired Wilcoxon tests, with heatmaps shown for visualization of pooled spot-level data only.

### Spatial autocorrelation analysis (Moran’s I)

To assess whether method disagreement exhibited spatial structure, we first calculated the variance of prediction scores across four methods for each spot and each cell type. Global Moran’s I was then computed for each sample-cell type combination using a k-nearest neighbor spatial weights matrix (k = 6). For pairwise analysis, the absolute difference between two methods’ predictions was calculated for each cell type per spot, and Moran’s I was computed on these difference values. Positive and significant Moran’s I values indicate spatial clustering of disagreement, while values near zero suggest random spatial distribution. For global Moran’s I, a k-nearest neighbor spatial weights matrix with k = 6 was used. This value was chosen based on the physical spacing of Visium spots, where each spot is approximately 55 μm in diameter with a center-to-center distance of about 100 μm, and k = 6 captures a local neighborhood of approximately 200–300 μm that is biologically relevant for local cell-cell interactions while providing stable autocorrelation estimates. Global Moran’s I values were computed independently for each sample, and sample-level Moran’s I values were used for cross-method comparisons using paired Wilcoxon tests with samples as paired observations.

### Local spatial autocorrelation analysis (LISA)

LISA analysis was performed to identify spots with significant local spatial clustering of tumor cells (defined as AT1-like, AT2-like, and Epi-like combined) or immune cells (B, T_NK, Mast, Myeloid, Plasma combined). For each sample and method, spots were binarized based on whether they were annotated as the cell lineage of interest. Local Moran’s I was calculated using a k-nearest neighbor weights matrix (k = 4). Spots were classified as High-High (HH; high value surrounded by high values), Low-Low, Outlier, or non-significant based on comparison to the global mean. LISA analysis was performed using a k-nearest neighbor weights matrix with k = 4, which captures the four immediate neighboring spots, representing the most direct local spatial unit for detecting significant spatial clustering at the single-spot resolution. The proportion of HH clusters was calculated per sample and used as the unit of analysis for cross-method Wilcoxon comparisons, ensuring that each sample contributed a single value per comparison.

### Neighborhood enrichment analysis

For each sample and method, spots were categorized into eight spatial groups: Tumor (AT1-like, AT2-like, Epi-like), B, T_NK, Mast, Myeloid, Plasma, Stroma, Endothelial, and Other. A k-nearest neighbor graph (k = 4) was constructed based on spatial coordinates. Observed adjacency counts between each pair of groups were tabulated and compared to expected counts from 50 random permutations of group labels. Z-scores were calculated as (observed - expected)/(standard deviation of permuted counts). Positive Z-scores indicate spatial attraction, negative Z-scores indicate spatial repulsion. A k-nearest neighbor graph with k = 4 was constructed based on spatial coordinates, using the four nearest spots to define local adjacency relationships, consistent with the spatial resolution of Visium data where each spot’s immediate neighbors represent the most biologically relevant local interactions. Z-scores were extracted per sample per method, and cross-method comparisons were performed on these sample-level Z-scores using paired Wilcoxon tests with samples as paired observations.

### Module score analysis

Module scores for TME cell types, tumor pathways, and TME functional pathways were calculated using the AddModuleScore function in Seurat. For TME cell type evaluation, canonical marker gene sets for T cells, B cells, plasma cells, myeloid cells, endothelial cells, stromal cells, and mast cells were defined from literature. For tumor pathway analysis, seven gene sets (Epithelial signature, Proliferative epithelial, EMT, MYC program, PI3K-AKT, MAPK-EGFR, UPR-ER-stress) were compiled from published sources. For TME functional pathway analysis, seven gene sets (Immune escape, ECM remodeling, Immune infiltration, Fibroblast activation, Antigen presentation, Angiogenesis, Hypoxia) were defined. Module scores were calculated on normalized spatial expression data, and scores were extracted for spots annotated as specific cell types (for TME cell scores) or as tumor cells (for tumor pathway scores) by each method. Mean module scores were calculated per sample per method by averaging spot-level scores within each sample, and all cross-method comparisons were performed on these sample-level means using paired Wilcoxon tests.

### Gene expression gradient analysis

To identify genes whose expression changes with distance to specific cell types, we performed a continuous differential expression analysis. For each sample, method, and target cell type, spatial coordinates of all spots annotated as that cell type were used as reference points. The Euclidean distance from each spot to its nearest reference cell was calculated. A linear model was then fit for each gene: expression ∼ distance + cell type (controlling for the spot’s own annotation), using log1p-transformed counts. Beta coefficients (gradient slopes) and p-values were extracted. Results were aggregated across samples for each method-cell type combination, and genes with FDR < 0.05 were considered significant. To identify robust biological signals, genes showing significance across at least two methods for a given cell type were retained. Heatmaps of mean beta coefficients and corresponding -log10 (p-values) were generated using ComplexHeatmap.

### Survival analysis

Kaplan-Meier survival analysis for selected gradient genes (CTSD, SLC34A2, FBP1, GDF15) was performed using the GEPIA2 online database (http://gepia2.cancer-pku.cn/#index). Lung adenocarcinoma patients were stratified into high and low expression groups using the 90% vs. 10% expression cutoff.

### Transcription factor inference

TF activity was inferred using the decoupleR framework with the DoRothEA/CollecTRI regulon database (human), applying the ULM (Univariate Linear Model) method to estimate regulatory activity scores for each transcription factor. This approach allowed systematic comparison of TF activity across cell types and annotation methods while accounting for variability introduced by spatial deconvolution strategies.

### Distance-associated differential pathway analysis

Distances between individual TME cells and tumor cells were calculated based on tissue coordinates. Within each of four major TME compartments, cells located within 300 spatial units of tumor cells were defined as tumor-proximal (“near”), while cells located beyond 1,000 units were defined as tumor-distal (“far”). Differential gene expression analysis comparing near versus far populations was independently performed for each cell type across all four annotation methods using Seurat’s FindMarkers framework. Genes were ranked by log2 fold change and subjected to Gene Set Enrichment Analysis (GSEA) using Gene Ontology Biological Process pathways. Consistently enriched or suppressed pathways were identified across all four annotation methods for each cell type. Mean normalized enrichment scores (NES) were calculated across methods, and pathway significance was summarized using adjusted p-values. For validation part, each selected pathway and their enrichment genes were used to construct module scores via AddModuleScore, and tumor versus ctrl groups were compared using Wilcoxon rank-sum tests.

### Statistical analysis

All statistical analyses were performed in R (version 4.5.1). Pairwise comparisons between methods for cell-type proportions, module scores, HH cluster ratios, and Z-scores were conducted using Wilcoxon rank-sum tests. For gradient analyses, Benjamini–Hochberg false discovery rate (FDR) correction was applied to p-values across genes. Visualization was performed using ggplot2, ComplexHeatmap, and patchwork.

### Software and package versions

R version 4.5.1; Seurat 5.4.0; CARD 1.1; spacexr 2.2.1; SPOTlight 1.12.2; ggplot2 4.0.2; dplyr 1.2.0; tidyr 1.3.2; ComplexHeatmap 2.24.1; circlize 0.4.16; patchwork 1.3.2; ggpubr 0.6.0; FNN 1.1.4.1; spdep 1.4.2; rstatix 0.7.2; decoupleR 2.14.0; clusterProfiler 4.16.0; msigdbr 24.1.0.

## Data Availability

The spatial transcriptomics data used in this study are publicly available and no new human/animal experiments were conducted. The 20 NSCLC tumor samples were obtained from Spatial transcriptomics (10x Visium) of human NSCLC. The 25 LUAD samples were obtained from GSE307534. The single-cell reference data were downloaded from the Human Cell Atlas Lung Biological Network (https://data.humancellatlas.org/hca-bio-networks/lung/atlases/lungv1-0). For independent validation, an additional single-cell RNA sequencing cohort comprising 22 samples (11 normal lung controls and 11 tumor samples), totaling over 88,000 cells, was analyzed from GSE131907. Process and analysis codes are available at https://github.com/sorrymaker03/LUAD_spatial.
